# Participant-reported effect of an Indigenous health continuing professional development initiative for specialists

**DOI:** 10.1186/s12909-021-02551-9

**Published:** 2021-02-18

**Authors:** Cheryl Barnabe, Raheem B. Kherani, Tom Appleton, Valerie Umaefulam, Rita Henderson, Lynden Crowshoe

**Affiliations:** 1 Departments of Medicine and Community Health Sciences, Cumming School of Medicine, University of Calgary, 3330 Hospital Dr NW, Calgary, AB T2N 4N1 Canada; 2 Department of Medicine, Faculty of Medicine, University of British Columbia, Richmond, BC Canada; 3 Department of Medicine, Schulich School of Medicine and Dentistry, Western University, London, ON Canada; 4 Department of Medicine, Cumming School of Medicine, University of Calgary, Calgary, AB Canada; 5 Department of Family Medicine, Cumming School of Medicine, University of Calgary, Calgary, AB Canada

**Keywords:** Indigenous populations, Arthritis, Continuing professional development, Cultural competency, Cultural safety

## Abstract

**Background:**

Health outcomes of Indigenous patients are impacted by culturally unsafe specialty care environments. The ‘Educating for Equity (E4E)’ program is a continuing professional development (CPD) intervention which incorporates skill-based teaching to improve Indigenous patient experiences and outcomes in healthcare interactions.

**Methods:**

The E4E program was delivered to rheumatologists in two phases, each delivered as experiential learning workshops where participants engaged with and applied course content within an interactive format focusing on real-time feedback. The phase 1 workshop focused on skill development of E4E Framework concepts and principles. Phase 2 concentrated on building capacity for teaching of E4E content. Evaluation of the program’s effectiveness was through longitudinal responses to the Social Cultural Confidence in Care Survey (SCCCS), self-reported strategies employed to address social issues and improve therapeutic relationships, engagement with teaching others, and satisfaction with the program.

**Results:**

Two cohorts of participants have participated in the program (*n* = 24 Phase 1, *n* = 10 Phase 2). For participants completing both phases of training, statistically significant improvements were observed in exploring social factors with patients, gaining knowledge and skills related to cultural aspects of care, improved communication and relationship building, and reflections on held stereotypes. Strategies to address social issues and build therapeutic relationships remained consistent throughout participation, while the training enhanced exploration and confidence to ask about cultural and traditional practices, and stronger communication strategies for exploring beliefs, expectations, social barriers, and residential school impacts on health. Participants reported feeling prepared to teach Indigenous health concepts to others and subsequently lead teaching with residents, fellows, and allied health professionals. Satisfaction with the delivery and content of the workshops was high, and participants valued interactions with peers in learning.

**Conclusions:**

This CPD intervention had a beneficial impact on self-reported confidence and enhanced practice strategies to engage with Indigenous patients.

**Supplementary Information:**

The online version contains supplementary material available at 10.1186/s12909-021-02551-9.

## Background

Arthritis conditions, both inflammatory and non-inflammatory, affect Indigenous Peoples in Canada to a greater extent than the general population. In addition to higher disease prevalence of nearly all rheumatic diseases [1], inequities in the social determinants of health among Indigenous people result in more adverse disease consequences [2, 3]. Related to historical legacies and the current nature of healthcare interactions with ongoing stereotyping and racism [4], Indigenous persons with arthritis have expressed that they are ‘toughing out’ arthritis rather than seeking longitudinal engagement with a rheumatologist [5]. This has broad implications for the provision of rheumatology specialty care, in which nearly three quarters of Canadian rheumatologists report providing some element of care to patients with an Indigenous identity [6]. Patient-proposed solutions include building specialty care environments that promote and practice culturally safe care [5]. Indigenous health education is mandated in Canadian undergraduate medical education programs [7], and the Royal College of Physicians and Surgeons of Canada also recently declared that it occur in specialty training [8]. These shifts are aligned with the Truth and Reconciliation Commission of Canada [9] Calls to Action to health professional schools for expanded training of practitioners, and they are supported by the Association of Faculties of Medical Schools [10]. Nevertheless, practicing subspecialty physicians are not mandated to complete such training. Necessary elements for training would be to acquire cultural competency, characterized by knowledge and awareness of Indigenous history and culture, being able to interact effectively with Indigenous people, and to practice cultural humility, which reflects growth and reflexivity while advocating for larger health system changes to reduce structural racism. Providing an effective platform for such learning is critical, not only for individual practice, but also for educators in the competence-by-design [11] era.

The Canadian Rheumatology Association (CRA) represents Canadian rheumatologists, with an approximate membership of 600. Beginning in 2016, a workshop on Indigenous health topics was incorporated within the Annual Scientific Meeting, but this lacked preparatory, practical, and longitudinal engagement from members. It was acknowledged that further actions would be required to ensure a solid knowledge base and practical skills application, with a national distribution of champions and culturally-competent providers that could ultimately improve outcomes for Indigenous patients across the country. In this initiative, we adapted the ‘Educating for Equity (E4E)’ [12] program, an evidence-based continuing professional development (CPD) initiative created for primary care physicians to support type 2 diabetes care with Indigenous patients, to the context of rheumatology specialty care.

Several publications related to the research informing the curriculum and results of the E4E program conducted in 3 primary care settings are published [13–15]. Briefly, the original E4E program and learning were designed to be problem-based, interactive, reflective, and in small groups. The workshop resulted in physicians becoming more confident in providing care to Indigenous patients with type 2 diabetes and modifying their approach to diabetes care when working with Indigenous patients. Impacts on the patient interaction were perceived, with physicians more frequently enquiring about patients’ socioeconomic conditions, increasing advocacy for social resources, becoming more skilled at eliciting patients’ use of or preference for culturally-based healing methods, and being more conscious of relationship-building with Indigenous patients using cultural factors. The physicians also gained cultural humility, as they perceived they were more self-reflective and aware of their stereotypes of Indigenous people [15]. Supported by this evidence, the E4E program was deemed to be an ideal CPD initiative to adapt to the needs of Canadian rheumatologists. To expand the impact of this workshop throughout the national rheumatology workforce, the CRA requested additional training components to enhance facilitation skills and to enable dissemination of competencies beyond the project phases; specifically that the initiative incorporated a ‘Train the Trainer’ approach. This article describes the adaptation, implementation, and physician education outcomes of the E4E CPD initiative for Canadian rheumatologists.

## Methods

### Initiative overview

**Fig. 1:**
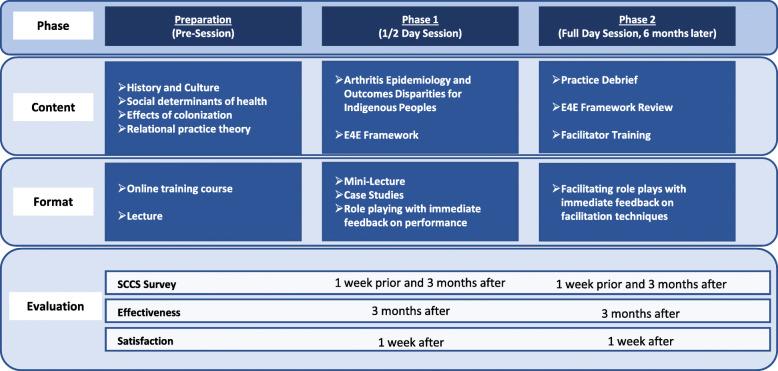
Canadian Rheumatology Association Indigenous Health Initiative

In collaboration with the E4E program lead (author LC) and team member (author RH), the Chairs of the CRA Operational Committees of Quality Care (author CB), Education (author RBK) and the Annual Scientific Meeting (author TA) undertook adaptation of the original program to create a two-phased program. While maintaining fidelity to the structure of the E4E workshops delivered to primary care physicians for diabetes care, case materials were adapted to the rheumatology specialty care context using qualitative data and patient narratives from prior research [5, 16]. Adaptation of the materials also drew on the clinical expertise of author CB (a Métis rheumatologist providing outreach clinical care to 3 First Nations and 1 urban Indigenous clinic in southern Alberta) and author LC (a First Nations primary care physician with extensive clinical experience in urban, rural and remote Indigenous communities), as well as the curriculum development and CPD expertise of author RBK. Figure 1 relays the phases of the CRA initiative, highlighting the format of training and the evaluation carried out at each phase.

#### Curriculum description

Phase 1: This half-day workshop provides content knowledge of the E4E Framework [12], and facilitates application of related skills through case based role-play. At this workshop, learners practice interviewing guided by the E4E Framework directives of engaging with the patient’s social reality and re-centring relationship. Using patient scripts providing context and motivation, one participant plays the patient, who is then interviewed by the other participant in an unscripted physician role. Midway through each case, the roles are switched. Role plays also begin to explore decision-making for rheumatoid arthritis therapy, and how cultural aspects may be engaged to support rheumatoid arthritis outcomes. Each patient-physician role play group is paired with a facilitator (E4E creator (LC), an E4E-immersed rheumatologist (CB) for Cohort 1, and in Cohort 2 additional facilitators trained in Cohort 1 (including RBK and TA)). Facilitators provided real-time feedback on learner performance, with feedback complemented by group sharing of promising approaches. Based on program feedback from Cohort 1, the didactic section of the workshop was shortened to allow additional time for role play and feedback, and an Elder (Mohawk Elder Amelia McGregor) participated in Cohort 2 activities.

Phase 2: Approximately 6 months after Phase 1, a full 1-day workshop was held. Participants shared experiences of applying the E4E Care Framework in their practices, and through further role plays solidified skills and approaches. However, the main objective of Phase 2 training was to develop facilitation skills to apply in teaching settings. In these sessions, small groups mirrored the phase 1 role-play interview format (scripted patient/unscripted physician dyad) but with the inclusion of one participant serving as the facilitator in giving feedback to the physician-learner. This facilitator-in-training received feedback on their facilitation skills from the session leaders (LC and CB). Following each small group session, the entire group also engaged in discussion around approaches to teaching in lectures, small group settings, and clinical encounters with trainees. Additional case stems were developed for Cohort 2 to ensure further practice opportunities. Session outlines are presented in Additional file 1: Appendix 1.

### Participant recruitment

An email invitation was distributed to CRA members by the organization’s secretariat to participate in the training program. Participants were requested to complete preparatory work to gain knowledge and understanding of Indigenous health context, including history of colonization and social determinants of health impacting health outcomes through online courses or local provincial health system materials. All participants attended a 1.5 h lecture on the E4E framework given during the Annual Scientific Meeting to the larger delegation, which demonstrated through didactic and practical applications how E4E may be applied in diabetes care. A prespecified maximum cohort size of 12 individuals was set to ensure feasibility and interactivity during the session; 2 additional participants were allowed for Cohort 2 as two additional facilitators (who completed both Phases of training in Cohort 1) were available. Those completing Phase 1 training were then invited to participate in a full-day Phase 2 training workshop to practice and solidify facilitation skills, with a commitment to deliver teaching sessions to their local residency programs and/or provide individual preceptorships to other rheumatologists. The workshops in both phases had a strategic regional representation to support distribution of the skillset and knowledge across the country.

### Evaluation

Modelled on the E4E study, our evaluation measured participant change in knowledge, attitude and approach to social and cultural factors related to healthcare with Indigenous patients with arthritis. The tool for tracking this, the ‘Social Cultural Confidence in Care Survey (SCCCS)’ [15] (presented in Additional file 1: Appendix 2) was developed from the E4E Framework and modifications to the Clinical Cultural Competency Questionnaire [17]. It is a 15 question Likert-scaled tool that includes questions on confidence in providing care to Indigenous patients, provider engagement with patient social and cultural factors, ability to facilitate relationships and to address inequity. Additionally, questions with free-text responses inquired about application of skills learned in the previous phase of training, specifically around approaches to addressing social issues and building therapeutic relationships with Indigenous patients (Phase 1) and teaching Indigenous health and E4E concepts to new learners (Phase 2). These items were distributed electronically for completion one week prior to and again 3 months after the completed workshop. Participant satisfaction with the content and delivery of the workshops, general feedback on the session content, and whether the workshops met the stated educational objectives were requested by electronic survey within 1 week of completion of each workshop. Participation in the evaluation was voluntary; Phase 2 Cohort 2 participant satisfaction surveys were regrettably not sent out due to a technical error, and also related to a technical error not all participant responses to the SCCCS could be linked to analyze change in responses over the duration of participation.

#### Analysis

Participant responses for the SCCCS were summarized for each phase of training. Changes in SCCCS responses for those who completed both Phase 1 and Phase 2 were assessed using the Wilcoxon signed rank test. Thematic analysis was used to identify and categorize the themes from participants’ text responses to open-ended questions around outcomes of participation. Session satisfaction was summarized descriptively.

#### Ethics

Approval for the study, recruitment strategy and evaluation was provided by the University of Calgary Conjoint Health Research Ethics Board (REB17–2465).

## Results

### Participants

A summary of number of participants and survey responses is provided in Table 1; no individual demographics were collected. In summary, 24 individuals participated in Phase 1 training, and 10 in Phase 2 training over the 2 years.

**Table 1:** Summary of Participation

	Cohort 1 (2018)	Cohort 2 (2019)
**Completed Pre-Phase 1 Evaluation**	6	8
**Attended Phase 1 Training Session**	10	14
**Completed Post-Phase 1 Evaluation**	8	*
**Completed Pre-Phase 2 Evaluation**	7	6
**Attended Phase 2 Training Session**	6	4
**Completed Post-Phase 2 Evaluation**	6	2

* Due to a technical issue with survey distribution, responses to the post-phase 1 evaluation are not available

### SCCCS responses

The frequency of individual responses and statistical significance of change for the 7 participants who completed both pre-Phase 1 and post-Phase 2 surveys is presented in Table 2. The workshops resulted in a significant change in several elements, including social factors (exploring with patients how stress, trauma, and recurrent adverse life experiences have potential impacts on arthritis outcomes, *p* = 0.02), all domains related to being culturally informed (knowledge about Indigenous healing traditions, *p* = 0.05; skill at eliciting patients’ use of and preferences for culture-based healing methods, *p* = 0.02; skill at providing culturally sensitive patient education and interventions, *p* = 0.02), facilitating relationships (effective communication skills, *p* = 0.05; employing cultural factors in approach to building a therapeutic relationship with Indigenous patients, *p* = 0.03), and addressing inequity (improving awareness of own stereotypes of Indigenous peoples, *p* = 0.03). All participant responses to the SCCCS are shown in Additional file 1: Appendix 3.

**Table 2:** Change in Social Cultural Confidence in Care: Completers of Both Phases of Training, with Pre-Phase 1 and Post-Phase 2 Survey Responses (*n* = 7)

	Pre-Phase 1					Post-Phase 2					Change
	Strongly Disagree	Disagree	Neutral	Agree	Strongly Agree	Strongly Disagree	Disagree	Neutral	Agree	Strongly Agree	Wilcoxon signed rank test
**GENERAL**											
Q1 – I am satisfied with my Indigenous patients’ clinical outcomes	1	1	2	3	0	0	4	0	3	3	1.00
Q2 – My level of confidence has improved with regards to providing care to Indigenous patients with arthritis	0	0	1	5	1	0	0	0	4	3	0.18
Q3 – I modify my arthritis care approach when working with Indigenous patients	0	0	0	4	3	0	1	0	1	5	0.70
**SOCIAL FACTORS**											
Q4 – When treating Indigenous patients with arthritis, I routinely and specifically inquire about socioeconomic conditions	0	1	2	0	4	0	0	2	4	1	0.79
Q5 – I explore with patients how stress, trauma and recurrent adverse life experiences have potential impacts on their arthritis outcomes	0	3	0	4	0	0	0	1	3	3	0.02
Q6 – I advocate for social resources that are key for my Indigenous patients with arthritis	0	0	1	2	4	0	0	0	5	2	0.65
**CULTURALLY INFORMED**											
Q7 -I am knowledgeable about Indigenous healing traditions	1	2	2	2	0	0	0	3	4	0	0.05
Q8 – I am skilled at eliciting patients’ use of and preferences for culture-based healing methods	1	2	2	2	0	0	0	1	4	2	0.02
Q9 – I am skilled at providing culturally sensitive patient education and interventions	0	3	2	2	0	0	0	2	3	2	0.02
**FACILITATING RELATIONSHIPS**											
Q10 – I am aware of my own cultural and professional identities	0	1	1	4	1	0	0	1	2	3	0.08
Q11 – I am an effective communicator with Indigenous patients	0	0	4	2	1	0	0	1	4	2	0.05
Q12 – I employ cultural factors in my approach to building a therapeutic relationship with Indigenous patients	0	3	3	0	1	0	0	1	2	4	0.03
**ADDRESSING INEQUITY**											
Q13 – I am knowledgeable of the impact of racism and prejudice in healthcare experienced by Indigenous populations	0	0	0	4	3	0	0	0	3	4	0.32
Q14 – I am aware of my own stereotypes of Indigenous peoples	0	0	1	6	0	0	0	0	3	4	0.03
Q15 – I have an understanding of colonization and its’ impact on Indigenous health outcomes	0	1	0	3	3	0	0	0	3	4	0.32

### Participation outcomes

Strategies for addressing social issues and enhancing therapeutic relationships remained consistent through the pre- and post-workshop reporting, with reinforcement of these provided through the program. As one participant reported, *“Social issues are at the heart of many challenges experienced by Indigenous patients. The training made this very clear and underscored the importance of leveraging these to develop a therapeutic alliance; Cohort 1, Participant 3”.* Rheumatologists remained comfortable asking directly about social factors, and assisting in identifying supports, whether this be from family, community, primary care system, or allied health providers. Several approaches they employed to build relationships with Indigenous patients were named, including flexibility in scheduling and availability to facilitate communication and provide care, having a non-judgmental approach, taking time to learn more about the individual and their beliefs, and the community they are from and live in. The participants welcomed collaborative decision making, involving family and supports in the process. The participants were engaged in serving as an advocate for the patient, acknowledged the legacy of historical events in patient interactions, and welcomed discussion around Indigenous health maintenance strategies in which patients may be engaged. The participants reported that the Phase 1 workshop provided enhanced communication strategies for exploring beliefs, expectations, social barriers and social determinants, traditional medicine, culture, residential school impacts, and previous healthcare experiences, as well as disease-related impacts of function and pain. They noted that based on what was learned of the E4E framework, they would, *“Open lines of communication beyond just medical issues earlier; Cohort 1, Participant 6”*, and had gained “*a better way to discuss beliefs, expectations and social barriers; Cohort 1, Participant 2”.* They were aware of the need to use a different approach in their interactions with Indigenous patients, including being attuned to the environment of the clinical interaction to ensure appropriate time and *“an open and comfortable environment for patients; Cohort 1, Participant 2”*, being present with the patient *“Observe, reflect, act; Cohort 2, Participant 3”*, and having humility and curiosity *“I must remain humble and curious and strive to enter more of a dialogue with my Indigenous patients; Cohort 2, Participant 1”*. Three months after the Phase 1 workshop, participants’ comments about how the workshop impacted, informed, or changed practice demonstrated growth in their skills, with reflexivity around their interactions and enhanced understanding of Indigenous patient realities. Interactions with colleagues in this learning was valued, as summarized by one participant: “*The workshop has provided opportunity to interact with colleagues from other regions and exchange experiences that help to reinforce the importance of learning indigenous culture, with social context in order to enhance care; Cohort 1, Participant 5”.*

Phase 2 participants reported commitment to providing Indigenous health content with trainees and office staff, perceiving that the facilitation training prepared them for teaching opportunities. Three months post-workshop, 5 out of 8 participants reported having led formal teaching sessions or clinical teaching in Indigenous health with rheumatology residents, fellows, and nurses. Benefit from participation in the initiative was reported, demonstrated by one participant’s feedback that it: “… *allowed me to more clearly articulate both concepts and approaches to care, including barriers to understanding and patient participation in chronic disease construct; Cohort 2, Participant 2”.*

### Satisfaction with training

All participants responded that the workshops met the stated objectives, were relevant to rheumatology, and met expectations. When asked to specify the most effective part of learning at the workshops, interactive discussions with peers, and case scenarios-based learning with role-playing and debriefing were stated by 6 participants each, as described here: “*Role playing and then debriefing. It allowed us to share strategies as clinicians, reflect on our strengths/weaknesses as communicators. Even more importantly, playing the “patient“ and understanding their social determinants behind their decisions was invaluable; Cohort 1, Participant 2”, and “Case scenarios based learning that used real life barriers and facilitated the integration of the information by playing the patient role; Cohort 1, Participant 6”*.

Time efficiency within the workshops was a recurrent theme, with a strong message to provide enough time for practice, discussion, demonstrations and examples. Feedback from Cohort 1 reinforced that case discussions needed to be rheumatology specific, thus additional focus away from diabetes case discussions was ensured for Cohort 2.

## Discussion

We share our experience of a CPD program for strengthening and enhancing physician skills in relationship building and communication strategies for interactions with Indigenous patients. Based on the survey and free-text responses from the evaluation, this program provided content knowledge, and was also seen to identify possible changes in practice and professional behavior in the delivery of high quality care to Indigenous patients. Case scenarios were illustrative and facilitated sharing between clinicians, reflecting on their strengths and weaknesses as communicators, and identifying helpful strategies for specific areas of interaction in rheumatology care. Perhaps more importantly, playing the “patient” role offered the opportunity to understand the relationship between social determinants, culture, and health decision making. These outcomes align with those reported from the E4E program for diabetes, wherein participants noted that group discussion and case studies were most effective in learning, with the workshop content improving participants’ understanding of diabetes social constructs and influence of social determinants of health [15]. The educational objectives set in our program were determined to have been achieved, with the program content and delivery meeting participant expectations, and with adequate opportunities to interact with peers and faculty. This collaborative approach between the CRA operational committees has also developed a blueprint for future initiatives to build resources offered to the membership.

Importantly, facilitation training was effective in the rapid engagement of participants in subsequently teaching medical trainees and allied health professionals. Strategic CRA member engagement from the distribution of geographic regions in Canada occurred, providing the organization with regional champions for Indigenous health who serve as a future resource to their colleagues and trainees. Indigenous health training opportunities have expanded in previous years, related to institutional responses to reconciliation and social accountability mandates of medical schools. Indeed, much of the curriculum is now delivered within medical schools, rather than residency programs or continuing professional development venues [18]. Many opportunities may focus on passive learning, providing content information related to the historical and continued legacy of colonization, or elements of cultural practice [19], recognized to be insufficient for preparation to work with Indigenous peoples [20], rather than providing transformative learning opportunities to ensure learners demonstrate core competencies to support Indigenous peoples’ health experiences [21]. Tenets of effective CPD, including establishing meaningful outcomes, applying effective teaching methods, and developing sessions to be interactive and engaging, enhancing participant self-awareness, promoting reflective practice, supporting interprofessional peer learning and lifelong learning, and requiring commitment to change statements must be upheld to support behavior change [22]. Meta-analysis review of CPD suggest that interactive interventions, multiple methods of interaction and small group work with a single discipline provide larger effect sizes [23]. This CPD program provides an example of an effective training program in Indigenous health.

We acknowledge limitations of this study. As stated in the methods section, there was an error in collecting workshop satisfaction responses after our Phase 2 training for our second year cohort, and not all participant responses to the SCCCS survey could be linked in the second year. Despite this, even with a limited cohort size, we were able to demonstrate significant changes in several domains of self-rated assessments. It was not feasible to collect clinical outcomes or experiences of Indigenous patients seen by the participants prior to and following the program, thus our findings are limited to describing the benefits to physician participants in the program. Demonstrating changes in patient experience and practice outcomes is desirable, and will be pursued in future offerings of the program. A relatively small number of physicians completed training, however coupled with family physicians trained in the E4E Framework in various provinces, this initiative provides the ability to scale the educational offering to other chronic disease management physicians. Recognizing that patients with chronic disease are at higher risk of admission to acute care facilities, we are also undertaking development of case materials to include common in-hospital scenarios as we expand the E4E training to other subspecialty groups.

## Conclusion

This CPD intervention is one approach in closing arthritis care gaps that are experienced by Indigenous patients. It had a beneficial impact on self-reported confidence and enhanced practice strategies to engage with Indigenous patients. The initiative resulted in beneficial interactions with similarly aligned colleagues and enhanced motivation to learn more about Indigenous health. These outcomes are desirable as we strive to achieve equity for Indigenous patients with arthritis conditions. Feedback allowed tailoring of session materials for length and detail, and employed teaching methods perceived as effective. Further studies can capture the effect of the intervention on long-term patient outcomes, processes of care, and the experience of patients, and expansion of the program to other disciplines.

## Supplementary Information

**Additional file 1: Appendix 1:** Rheumatology Indigenous Health Initiative Curriculum. **Appendix 2:** Evaluation Package. **Appendix 3.** All responses to the Social Cultural Confidence in Care Survey

## Data Availability

The datasets generated during and analysed during the current study are not publicly available due to our ethics board requiring these to be held securely by members of the research team, but aggregate data are available from the corresponding author on reasonable request.
