# Supplementary material for: Participant-reported effect of an Indigenous health continuing professional development initiative for specialists
Source: BMC Med Educ. 2021 Feb 18;21:116. doi: 10.1186/s12909-021-02551-9 (PMC7891014; doi:10.1186/s12909-021-02551-9)
Supplement: Supplementary file 1 — Additional file 1: Appendix 1: Rheumatology Indigenous Health Initiative Curriculum. Appendix 2: Evaluation Package. Appendix 3. All responses to the Social Cultural Confidence in Care Survey [file 12909_2021_2551_MOESM1_ESM.docx]

Supplemental Material for:

**Participant-Reported Effect of an Indigenous Health Continuing Professional Development Initiative for Specialists**

Authors: Cheryl Barnabe, MD, MSc; Raheem B Kherani, MD, MHPE; Tom Appleton, MD, PhD; Valerie Umaefulam, PhD; Rita Henderson, PhD; and Lynden Crowshoe, MD

**Appendix 1:** **Rheumatology Indigenous Health Initiative Curriculum**

**Phase 1 Workshop Guide**

**Overview:** This three hour session will explore social and cultural dimensions within the clinical management of arthritis with Indigenous people. This session will contextualize the theoretical constructs of Cultural Competency, Cultural Safety and Structural Competency within a Patient-Centred Approach frame. The session will provide an opportunity for participants to engage in an in-depth and critical examination of social determinants shaped by a Canadian colonial history that continues to influence Indigenous patients’ experiences of RA and the physician’s ability to provide effective care.

**Educational Content and Methods:** The session’s case-based content originates from qualitative research themes and existing peer reviewed literature. Exploration of each case will be through an existing framework, ‘Educating for Equity’, that illustrates the unique pathways that link inequity and the social determinants of health to chronic disease for Indigenous populations. This framework provides specific clinical recommendations for integrating social and cultural factors shown to enhance patient care and disease outcomes. The session will employ a small group practice-based learning approach to encourage an interactive learning mode framed through key questions that will enable learners in their comprehension and integration of conceptual and applied knowledge related to each case.

**Educational Objectives:** At the conclusion of this session, in regards to Indigenous patients with arthritis, participants will be able to demonstrate:

1. Chronic disease management that engages with key social factors facilitated by culturally informed approaches to supporting patients
2. Therapeutic relationship building that incorporates culturally attuned approaches and address discord arising from health care inequity

**Agenda**

1. Session start
2. Opening by Elder
3. Session overview of content, methods and outcomes
4. Introductions
5. Discussion: *What are your challenges, barriers and successes in providing care to Indigenous patients with RA?*
6. RA case presentation (Terry, Part 1)
   1. Discussion: *As you review the case, identify and reflect on social realities that influence RA outcomes and clinical relationship issues that influence care.’*
7. Approach to incorporating social and cultural domains into clinical practice: Overview of the ‘Educating for Equity’ model
8. Small groups and facilitated questions: RA case revisited

Terry, Part 1: *In this first round of role play, the aim is to provide feedback to the person playing the physician role how they should establish relationship with Terry, and how to elicit the social realities influencing his decision making with regards to his RA follow-up and therapy.*

Terry, Part 2: *Small groups and facilitated questions. In this second round of role play, the aim is to provide feedback to the person playing the physician role how they should establish relationship with Terry, and how to elicit the cultural themes and approaches that can support Terry’s care.*

1. Summary and participant feedback

**Phase 2 Workshop Guide**

**Overview:** This 1 day session will provide an opportunity for participants to engage in developing facilitation skills for interacting with Indigenous patients, while reinforcing concepts and approaches of the ‘Educating for Equity’ Framework.

**Educational Content and Methods:** The session will employ a small group practice-based learning approach to encourage an interactive learning mode framed through individualized feedback on facilitation skills. The session’s case-based content originates from qualitative research themes and existing peer reviewed literature. Exploration of each case will be through an existing framework, ‘Educating for Equity’, that illustrates the unique pathways that link inequity and the social determinants of health to chronic disease for Indigenous populations. This framework provides specific clinical recommendations for integrating social and cultural factors shown to enhance patient care and disease outcomes.

**Educational Objectives:** At the conclusion of this session, participants will be able to demonstrate:

1. An ability to provide feedback and guidance to physicians interacting with Indigenous patients.

Additionally, in regards to Indigenous patients with arthritis, participants will be able to demonstrate:

2. Chronic disease management that engages with key social factors facilitated by culturally informed approaches to supporting patients

3. Therapeutic relationship building that incorporates culturally attuned approaches and address discord arising from health care inequity

**Agenda:**

- - 1. Session start
  1. Introduction to the day
  2. Debrief on patient interactions and explorations since Phase 1 session
     1. Educating for Equity Framework review and exploration
     2. Simulation: Lecture presentation
     3. Small groups and facilitator practice: Interactions 1-8
     4. Discussion about other teaching formats (Entrustable professional activities, journal clubs)
     5. Summary and participant feedback

**Appendix 2: Evaluation Package**

**Social Cultural Confidence in Care Survey (SCCCS)**

Note that the SCCCS is completed at each Phase Pre-Assessment (1 week prior) and Post-Assessment (3 months post-workshop). Participants rank each question on a Likert Scale: Strongly Disagree, Disagree, Neutral, Agree, Strongly Agree

| **General** |
| --- |
| 1. I am satisfied with my Indigenous patients’ clinical outcomes. |
| 1. My level of confidence has improved with regards to providing care to Indigenous patients with arthritis. |
| 1. I modify my arthritis care approach with working with Indigenous patients. |
| **Social Factors** |
| 1. When treating Indigenous patients with arthritis, I routinely and specifically enquire about socioeconomic conditions (e.g. income, employment, education, food security, transportation, housing, access to services) |
| 1. I explore with patients how stress, trauma, and recurrent adverse life experiences have potential impacts on their arthritis outcomes. |
| 1. I advocate for social resources that are key for my Indigenous patients with arthritis. |
| **Culturally Informed** |
| 1. I am knowledgeable about Indigenous healing traditions. |
| 1. *I am skilled at eliciting patients’ use of and preferences for culture-based healing methods. |
| 1. *I am skilled at providing culturally sensitive patient education and interventions. |
| **Facilitating Relationships** |
| 1. *I am aware of my own cultural and professional identities. |
| 1. I am an effective communication with Indigenous patients. |
| 1. I employ cultural factors in my approach to building a therapeutic relationship with Indigenous patients. |
| **Addressing Inequity** |
| 1. *I am knowledgeable of the impact of racism and prejudice in healthcare experienced by Indigenous populations. |
| 1. *I am aware of my own stereotypes of Indigenous peoples. |
| 1. I have an understanding of colonization and its impact on Indigenous health outcomes. |

**Phase 1: Pre-Workshop Needs Assessment and Reflection Activity**

1. How do you address social issues that impact arthritis outcomes for Indigenous patients? Briefly list 3 of your strategies.
2. What is your approach to enhancing therapeutic relationships with your Indigenous patients with arthritis? Briefly list 3 of your key strategies.
3. Please list 3 aspects of your approach to arthritis management in Indigenous patients that you would like to get feedback on during the workshop.

**Phase 1: Post-Workshop Reflection Activity**

1. Describe 2 ways that the workshop has impacted, informed or changed your practice and what are the outcomes?
2. How do you address social issues that impact arthritis outcomes for Indigenous patients? Briefly list 3 of your key strategies.

3. What is your approach to enhancing therapeutic relationships with your Indigenous patients with arthritis? Briefly list 3 of your key strategies.

**Phase 2: Pre-Workshop Needs Assessment and Reflection Activity**

1. Have you led group learning in rheumatology before? Briefly list what types of sessions you have facilitated, and the audience (e.g. practicing physicians, residents, allied health, etc).
2. Have you led group learning in Indigenous health before? Briefly list what types of sessions you have facilitated, and the audience (e.g. practicing physicians, residents, allied health, etc).
3. Have you led individual learning in rheumatology before? Briefly list what types of sessions you have facilitated, and the audience (e.g. practicing physicians, residents, allied health, etc).
4. Have you led individual learning in Indigenous health before? Briefly list what types of sessions you have facilitated, and the audience (e.g. practicing physicians, residents, allied health, etc).
5. Please list what skills you hope to acquire during the workshop.

**Phase 2: Post-Workshop Reflection Activity**

1. Since you completed the workshop, have you had the opportunity to provide teaching in Indigenous health? Please list what sessions you have led.
2. Do you feel the training you received prepared you adequately for these teaching opportunities? Please provide which aspects were well supported, and which areas you feel still need additional support.
3. Did this Phase 2 training further enhance your skills in developing therapeutic relationships with Indigenous patients? Please briefly explain.
4. Did the Phase 2 training further enhance your skills in addressing social issues that your Indigenous patients encounter? Please briefly explain.

**Post-Workshop Program Evaluation:** Completed within 1 week of Phase 1 and Phase 2 workshops

**Educational Objectives**

| The program met the stated objectives of improving physician capacity to: | Strongly Agree | Neutral | Strongly Disagree |
| --- | --- | --- | --- |
| 1. Describe key social factors that affect Indigenous arthritis outcomes. | 5 4 3 2 1 | | |
| 1. Describe and demonstrate culturally attuned approaches to building therapeutic relationships with Indigenous arthritis patients. | 5 4 3 2 1 | | |
| 1. Identify and demonstrate methods to address discord in the doctor-patient relationship stemming from inequality and marginalization of Indigenous people in health care and society. | 5 4 3 2 1 | | |
| 1. Describe and demonstrate culturally informed ways to support Indigenous arthritis patients. | 5 4 3 2 1 | | |

**Program Content and Delivery**

|  | Strongly Agree | Neutral | Strongly Disagree |
| --- | --- | --- | --- |
| 1. The program content was relevant to rheumatology. | 5 4 3 2 1 | | |
| 1. The program met my expectations. | 5 4 3 2 1 | | |
| 1. The program was well organized. | 5 4 3 2 1 | | |
| 1. Disclosure of potential conflicts of interest was clearly communicated. | 5 4 3 2 1 | | |
| 1. Faculty members were effective in delivering/facilitating the program. | 5 4 3 2 1 | | |
| 1. There were adequate opportunities to interact with my peers. | 5 4 3 2 1 | | |
| 1. There were adequate opportunities to interact with program faculty. | 5 4 3 2 1 | | |
| 1. The information I learned will be used in my future practice. | 5 4 3 2 1 | | |

What was the most effective part of the program? Why?

What was the least effective part of this program? Why?

Based on what you learned of the E4E framework, describe two ways in which you will change your practice

What must you do to integrate these decisions into your practice?

What kinds of barriers do you foresee?

General Comments:

**Appendix 3. All responses to the Social Cultural Confidence in Care Survey**

**PHASE 1**

|  | **Pre-Phase 1 n=14** | | | | | **Post-Phase 1 n=5** | | | | |
| --- | --- | --- | --- | --- | --- | --- | --- | --- | --- | --- |
|  | Strongly Disagree | Disagree | Neutral | Agree | Strongly Agree | Strongly Disagree | Disagree | Neutral | Agree | Strongly Agree |
| **GENERAL** | | | | | | | | | | |
| Q1 – I am satisfied with my Indigenous patients’ clinical outcomes | 1 (7%) | 3 (21%) | 5 (36%) | 5 (36%) | 0 (0%) | 0 (0%) | 0 (0%) | 1 (20%) | 4 (80%) | 0 (0%) |
| Q2 – My level of confidence has improved with regards to providing care to Indigenous patients with arthritis | 0 (0%) | 1 (7%) | 3 (21%) | 9 (64%) | 1 (7%) | 0 (0%) | 0 (0%) | 1 (20%) | 2 (40%) | 2 (40%) |
| Q3 – I modify my arthritis care approach when working with Indigenous patients | 0 (0%) | 1 (7%) | 2 (14%) | 7 (50%) | 3 (29%) | 0 (0%) | 0 (0%) | 1 (20%) | 2 (40%) | 2 (40%) |
| **SOCIAL FACTORS** | | | | | | | | | | |
| Q4 – When treating Indigenous patients with arthritis, I routinely and specifically inquire about socioeconomic conditions | 0 (0%) | 1 (7%) | 6 (43%) | 2 (14%) | 5 (36%) | 0 (0%) | 0 (0%) | 0 (0%) | 5 (100%) | 0 (0%) |
| Q5 – I explore with patients how stress, trauma and recurrent adverse life experiences have potential impacts on their arthritis outcomes | 0 (0%) | 3 (21%) | 2 (14%) | 8 (57%) | 1 (7%) | 0 (0%) | 0 (0%) | 1 (20%) | 3 (60%) | 1 (20%) |
| Q6 – I advocate for social resources that are key for my Indigenous patients with arthritis | 0 (0%) | 1 (7%) | 3 (21%) | 3 (21%) | 7 (50%) | 0 (0%) | 1 (20%) | 2 (40%) | 0 (0%) | 2 (40%) |
| **CULTURALLY INFORMED** | | | | | | | | | | |
| Q7 -I am knowledgeable about Indigenous healing traditions | 3 (21%) | 3 (21%) | 4 (29%) | 4 (29%) | 0 (0%) | 0 (0%) | 2 (40%) | 1 (20%) | 2 (40%) | 0 (0%) |
| Q8 – I am skilled at eliciting patients’ use of and preferences for culture-based healing methods | 2 (14%) | 5 (36%) | 5 (36%) | 2 (14%) | 0 (0%) | 0 (0%) | 2 (40%) | 1 (20%) | 2 (40%) | 0 (0%) |
| Q9 – I am skilled at providing culturally sensitive patient education and interventions | 1 (7%) | 5 (36%) | 5 (36%) | 3 (21%) | 0 (0%) | 0 (0%) | 2 (40%) | 1 (20%) | 1 (20%) | 1 (20%) |
| **FACILITATING RELATIONSHIPS** | | | | | | | | | | |
| Q10 – I am aware of my own cultural and professional identities | 0 (0%) | 1 (7%) | 3 (21%) | 9 (64%) | 1 (7%) | 0 (0%) | 0 (0%) | 0 (0%) | 3 (60%) | 2 (40%) |
| Q11 – I am an effective communicator with Indigenous patients | 1 (7%) | 0 (0%) | 6 (43%) | 6 (43%) | 1 (7%) | 0 (0%) | 0 (0%) | 1 (20%) | 3 (60%) | 1 (20%) |
| Q12 – I employ cultural factors in my approach to building a therapeutic relationship with Indigenous patients | 1 (7%) | 4 (29%) | 1 (7%) | 7 (50%) | 1 (7%) | 0 (0%) | 1 (20%) | 0 (0%) | 2 (40%) | 2 (40%) |
| **ADDRESSING INEQUITY** | | | | | | | | | | |
| Q13 – I am knowledgeable of the impact of racism and prejudice in healthcare experienced by Indigenous populations | 0 (0%) | 0 (0%) | 0 (0%) | 9 (64%) | 5 (36%) | 0 (0%) | 0 (0%) | 0 (0%) | 2 (40%) | 3 (60%) |
| Q14 – I am aware of my own stereotypes of Indigenous peoples | 0 (0%) | 0 (0%) | 1 (7%) | 13 (93%) | 0 (0%) | 0 (0%) | 0 (0%) | 0 (0%) | 4 (80%) | 1 (20%) |
| Q15 – I have an understanding of colonization and its’ impact on Indigenous health outcomes | 0 (0%) | 2 (14%) | 1 (7%) | 6 (43%) | 5 (36%) | 0 (0%) | 0 (0%) | 0 (0%) | 2 (40%) | 3 (60%) |

**PHASE 2**

|  | **Pre-Phase 2 n=13** | | | | | **Post-Phase 2 n=8** | | | | |
| --- | --- | --- | --- | --- | --- | --- | --- | --- | --- | --- |
|  | Strongly Disagree | Disagree | Neutral | Agree | Strongly Agree | Strongly Disagree | Disagree | Neutral | Agree | Strongly Agree |
| **GENERAL** | | | | | | | | | | |
| Q1 – I am satisfied with my Indigenous patients’ clinical outcomes | 3 (23%) | 3 (23%) | 2 (15%) | 5 (39%) | 0 (0%) | 0 (0%) | 4 (50%) | 0 (0%) | 3 (37.5%) | 1 (12.5%) |
| Q2 – My level of confidence has improved with regards to providing care to Indigenous patients with arthritis | 0 (0%) | 2 (15%) | 1 (8%) | 6 (46%) | 4 (31%) | 0 (0%) | 0 (0%) | 0 (0%) | 5 (62.5%) | 3 (37.5%) |
| Q3 – I modify my arthritis care approach when working with Indigenous patients | 0 (0%) | 0 (0%) | 3 (23%) | 7 (54%) | 3 (23%) | 0 (0%) | 1 (12.5%) | 1 (12.5%) | 1 (12.5%) | 5 (62.5%) |
| **SOCIAL FACTORS** | | | | | | | | | | |
| Q4 – When treating Indigenous patients with arthritis, I routinely and specifically inquire about socioeconomic conditions | 0 (0%) | 1 (8%) | 2 (15%) | 7 (54%) | 3 (23%) | 0 (0%) | 0 (0%) | 2 (25%) | 5 (62.5%) | 1 (12.5%) |
| Q5 – I explore with patients how stress, trauma and recurrent adverse life experiences have potential impacts on their arthritis outcomes | 0 (0%) | 0 (0%) | 4 (31%) | 6 (46%) | 3 (23%) | 0 (0%) | 0 (0%) | 1 (12.5%) | 4 (50%) | 3 (37.5%) |
| Q6 – I advocate for social resources that are key for my Indigenous patients with arthritis | 0 (0%) | 0 (0%) | 2 (15%) | 8 (62%) | 3 (23%) | 0 (0%) | 0 (0%) | 0 (0%) | 5 (62.5%) | 3 (37.5%) |
| **CULTURALLY INFORMED** | | | | | | | | | | |
| Q7 -I am knowledgeable about Indigenous healing traditions | 1 (8%) | 3 (23%) | 6 (46%) | 2 (15%) | 1 (8%) | 0 (0%) | 0 (0%) | 3 (37.5%) | 5 (62.5%) | 0 (0%) |
| Q8 – I am skilled at eliciting patients’ use of and preferences for culture-based healing methods | 0 (0%) | 5 (39%) | 4 (31%) | 4 (31%) | 0 (0%) | 0 (0%) | 0 (0%) | 2 (25%) | 4 (50%) | 2 (25%) |
| Q9 – I am skilled at providing culturally sensitive patient education and interventions | 0 (0%) | 4 (31%) | 3 (23%) | 5 (39%) | 1 (8%) | 0 (0%) | 0 (0%) | 2 (25%) | 4 (50%) | 2 (25%) |
| **FACILITATING RELATIONSHIPS** | | | | | | | | | | |
| Q10 – I am aware of my own cultural and professional identities | 0 (0%) | 0 (0%) | 3 (23%) | 7 (54%) | 3 (23%) | 0 (0%) | 0 (0%) | 1 (14%) | 3 (43%) | 3 (43%) |
| Q11 – I am an effective communicator with Indigenous patients | 1 (8%) | 0 (0%) | 6 (46%) | 4 (31%) | 2 (15%) | 0 (0%) | 0 (0%) | 1 (12.5%) | 4 (50%) | 3 (37.5%) |
| Q12 – I employ cultural factors in my approach to building a therapeutic relationship with Indigenous patients | 0 (0%) | 0 (0%) | 6 (46%) | 6 (46%) | 1 (8%) | 0 (0%) | 0 (0%) | 1 (12.5%) | 3 (37.5%) | 4 (50%) |
| **ADDRESSING INEQUITY** | | | | | | | | | | |
| Q13 – I am knowledgeable of the impact of racism and prejudice in healthcare experienced by Indigenous populations | 0 (0%) | 1 (8%) | 0 (0%) | 6 (46%) | 6 (46%) | 0 (0%) | 0 (0%) | 0 (0%) | 4 (50%) | 4 (50%) |
| Q14 – I am aware of my own stereotypes of Indigenous peoples | 1 (8%) | 0 (0%) | 1 (8%) | 8 (62%) | 3 (23%) | 0 (0%) | 0 (0%) | 0 (0%) | 4 (50%) | 4 (50%) |
| Q15 – I have an understanding of colonization and its’ impact on Indigenous health outcomes | 0 (0%) | 1 (8%) | 2 (15%) | 6 (46%) | 4 (31%) | 0 (0%) | 0 (0%) | 0 (0%) | 4 (50%) | 4 (50%) |
